# Clinician Knowledge of Chagas Disease After an Educational Intervention

**DOI:** 10.1001/jamanetworkopen.2024.19906

**Published:** 2024-07-02

**Authors:** Kerlly J. Bernabé, Eric Dumonteil, Claudia Herrera

**Affiliations:** 1 Department of Tropical Medicine and Infectious Disease, School of Public Health and Tropical Medicine, and Vector-Borne and Infectious Disease Research Center, Tulane University, New Orleans, Louisiana

## Abstract

This survey study describes the self-reported medical knowledge among physicians before and after attending a lecture on Chagas disease.

## Introduction

Chagas disease is a major neglected parasitic infection, affecting an estimated 287 711 people in the US.^1^ Delayed diagnosis and treatment is associated with increased risk of cardiac, gastrointestinal, and neurological alterations with severe morbidity and mortality.^2^ Chagas disease remains underdiagnosed (<1%)^3^ and untreated (<0.3%)^4^ due to structural, psychosocial, clinical, and systemic barriers and disproportionately affects Hispanic or Latino populations in the US.^5^ Limited efforts exist to educate physicians on this disease; thus, patient screening is lacking. We evaluated the extent of Chagas disease testing and measured medical knowledge before and after a training lecture.

## Methods

In this survey study, the number of Chagas disease tests ordered in major hospitals in New Orleans, including Tulane Medical Center (TMC), University Medical Center (UMC), and Veterans Affair Medical Center (VAMC), was ascertained using electronic health records (EHRs). Patient screening is described in eMethods in Supplement 1. To assess medical knowledge, anonymous cross-sectional surveys were administered to physicians from multiple medical specialties (eTable in Supplement 1) before and after training. The Tulane Institutional Review Board deemed this survey study exempt from review and waived informed consent because it was not human participant research. We followed the AAPOR reporting guideline.

Frequencies were computed for demographics. Survey responses before and after training were compared using McNemar χ^2^ tests. Association between physician characteristics and knowledge was evaluated using χ^2^ tests. Results were cross-tabulated and reported as counts. Two-sided *P* < .05 indicated statistical significance. Data analysis was performed with R 4.3.1 (R Core Team).

## Results

Between 2007 and 2023, 1242 tests were ordered at TMC, 48 at UMC, and 6 at VAMC. At TMC, 30 of 1242 (2%) screened were Hispanic or Latino patients, 385 (31%) were transplant recipients or donors, 326 (26%) had cancer, 199 (16%) had preoperative examinations or substance use disorder, and 11 (<1%) had a diagnosis compatible with Chagas disease. Six adults (0.5%) and 1 pediatric patient (0.1%) had positive test results; 4 of these were Hispanic or Latino patients and 1 had a diagnosis compatible with Chagas disease. At UMC, 36 of 48 (75%) screened identified as Hispanic or Latino patients, and 28 (58%) had a diagnosis compatible with Chagas disease. No pregnant patient was screened.

Of 474 physicians who attended the Chagas disease lecture, 280 (59%) completed the baseline survey and 181 of 280 (65%) completed the posttraining survey, and represented various medical specialties, experience levels, races, and ethnicities (Table). At baseline, physicians had adequate general knowledge of Chagas disease but lower percentages of correct answers for aspects such as transmission routes (56%); diagnostic methods (25%); treatment for adult, pediatric, or pregnant patients (17%-49%); disease reactivation (32%); and recognition of Chagas cardiomyopathy (20%-40%) (Figure). Baseline knowledge was higher for clinicians with prior vs without training (345 [65%] vs 422 [51%]), and significantly differed among medical specialties (eg, cardiology: 82 [61%]; pediatrics: 79 [40%]) (Table). Experience level, race and ethnicity, and estimated proportion of Hispanic or Latino patients had no association with Chagas disease knowledge. After training, knowledge significantly increased (>82% correct answers), except for clinical manifestations in infants (28%). Insufficient knowledge remained regarding disease reactivation (45%), diagnostics (75%), and congenital risk (68%). Physicians expressed concerns about access to rapid screening, performance of tests, and algorithm to confirm diagnosis.

**Table.** Associations Between Participant Characteristics and Baseline Knowledge

Characteristic	Participants, No. (%) (N = 154)	Questions, No./total No. (%)		SE	χ^2^ test	*df*	*P* value
		Correct	Incorrect				
Prior training							
No	94 (61)	422/830 (51)	408/830 (49)	1.7	25.0	1	<.001
Yes	60 (39)	345/533 (65)	188/533 (35)	2.1			
Race and ethnicity^a^							
Asian	26 (17)	142/231 (61)	89/231 (39)	3.2	8.0	4	.10
Black	8 (5)	47/72 (65)	25/72 (35)	5.6			
Hispanic or Latino	13 (9)	68/114 (60)	46/114 (40)	4.6			
White	91 (61)	431/804 (54)	373/804 (46)	1.8			
2 Racial and ethnic groups	11 (7)	56/98 (57)	42/98 (43)	5.0			
Specialty							
Cardiology	15 (10)	82/135 (61)	53/135 (39)	4.2	37.0	4	<.001
Family medicine	24 (16)	121/216 (56)	95/216 (44)	3.4			
Internal medicine	66 (43)	375/596 (63)	221/596 (37)	2.0			
Obstetrics and gynecology	24 (16)	110/216 (51)	106/216 (49)	3.4			
Pediatrics	25 (16)	79/200 (40)	121/200 (60)	3.5			
Years of experience^b^							
>10	29 (62)	126/250 (50)	124/250 (50)	3.2	1.21	1	.27
≤10	18 (38)	89/159 (56)	70/159 (44)	3.9			
Percentage of patient population with Hispanic, Latino, or Spanish origin^c^							
<1	3 (2)	10/25 (40)	15/25 (60)	9.8	5.0	1	.29
1-10	39 (30)	209/347 (60)	138/347 (40)	2.6			
11-25	60 (47)	297/532 (56)	235/532 (44)	2.2			
26-50	20 (16)	105/179 (59)	74/179 (41)	3.7			
>50	6 (5)	28/51 (55)	23/51 (45)	7.0			

^a^ Numbers do not sum to 154; participants who preferred not to answer this question were excluded. Race and ethnicity were self-identified in surveys.

^b^ Numbers do not sum to 154; only attending physicians, nurse practitioners, and registered nurses answered this question.

^c^ Numbers do not sum to 154; participants who responded “I do not know” to this question were excluded.

**Figure.**
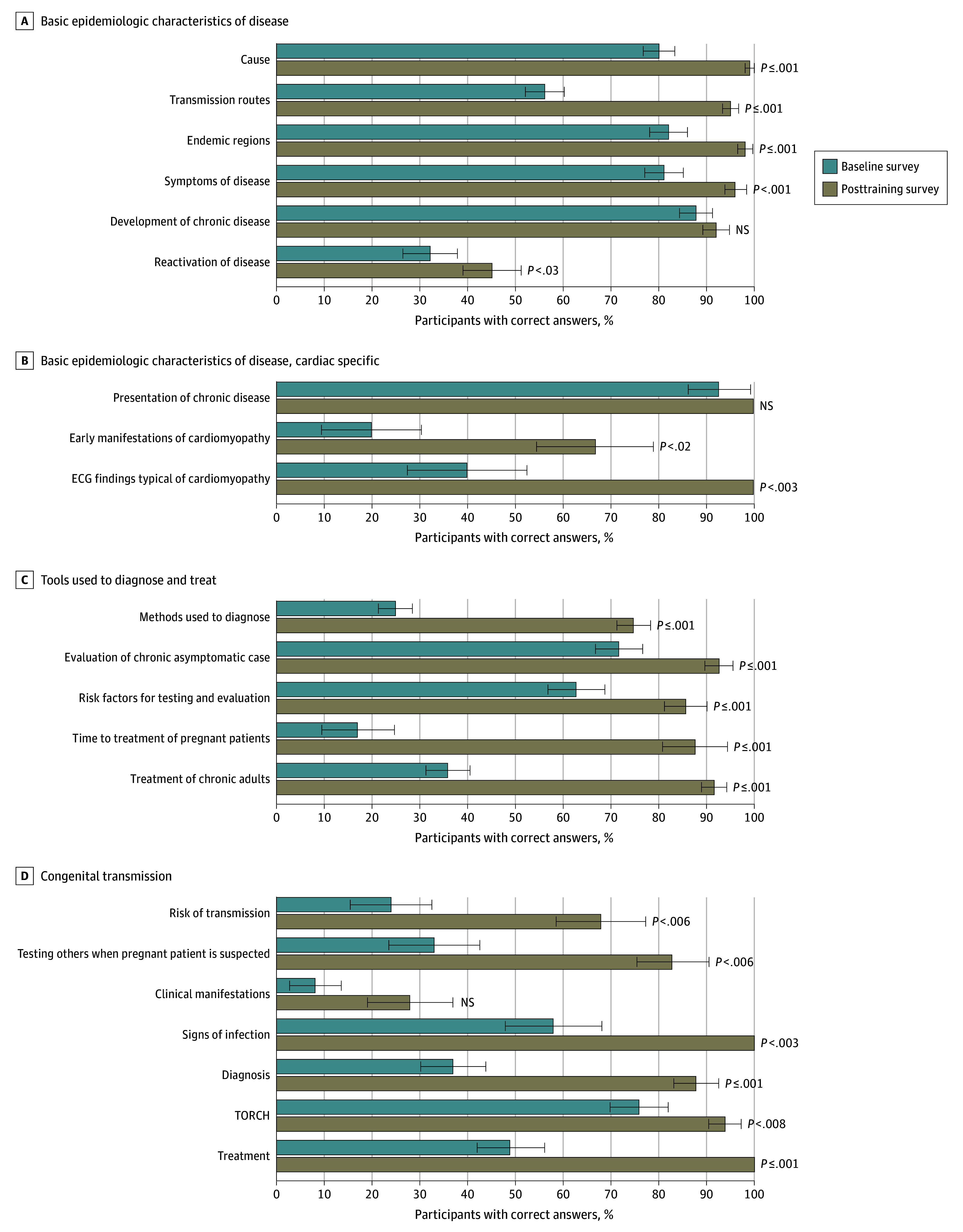
Clinician Knowledge of Chagas Disease at Baseline and After Training Error bars represent SEs. ECG indicates electrocardiogram; NS, not significant; TORCH, toxoplasmosis, other agents (syphilis, hepatitis B), rubella, cytomegalovirus, and herpes simplex.

## Discussion

Current testing for Chagas disease is either targeted and limited in scale or missing most at-risk populations, resulting in low detection of cases. Improved screening is needed, particularly for young adult, pregnant, and pediatric patients who would most benefit from early diagnosis and treatment.^6^

Limited knowledge of Chagas disease restricts clinicians’ ability to offer optimum patient screening and care. A study limitation is that selection bias can only be inferred because the demographics of nonparticipants were not collected.

Our findings indicate that continued medical education can mitigate knowledge gaps on Chagas disease, at least in the short-term. Educating physicians can potentially change clinical practice to one with more screening, better care, and less health disparities for patients with Chagas disease.

## Notes

Supplement 1.
eMethods.
**eTable.** Survey Questions

Supplement 2.
Data Sharing Statement
