# Supplementary material for: Clinician Knowledge of Chagas Disease After an Educational Intervention
Source: JAMA Netw Open. 2024 Jul 2;7(7):e2419906. doi: 10.1001/jamanetworkopen.2024.19906 (PMC11220560; doi:10.1001/jamanetworkopen.2024.19906)
Supplement: Supplement 1. — eMethods. eTable. Survey Questions [file jamanetwopen-e2419906-s001.pdf]

Supplemental Online Content

Bernabé KJ, Dumonteil E, Herrera C. Clinician knowledge of Chagas disease after an educational intervention. *JAMA Netw Open*. 2024;7(7):e2419906.
doi:10.1001/jamanetworkopen.2024.19906

eMethods.

eTable. Survey Questions

This supplemental material has been provided by the authors to give readers additional information about their work.

© 2024 Bernabé KJ et al. *JAMA Network Open*

eMethods.

Study Design
We determined the number of lab orders for Chagas disease serological testing placed at major hospitals in recent years by querying electronic health records (EHR) systems for de-identified data and analyzed the demographic profile of patients tested. We also prospectively assessed the knowledge and awareness of Chagas disease from healthcare professionals from multiple institutions. Study participants were recruited among attendees to grand rounds, didactic lectures, and multi-institutional conferences on Chagas disease. Attendees were invited to participate in an anonymous survey on the disease before and after receiving the training.

Chagas Disease Testing
EHR from Tulane Medical Center (TMC), University Medical Center (UMC) and New Orleans Veterans Affair Medical Center (VA) were queried for test orders on *T. cruzi* infection/Chagas disease. Since this study was anonymous, test orders were not linked to healthcare professionals or institutions. The system queried at TMC was electronic ClinicalWorks (eClinicalWorks) for outpatient care between January 2007 to mid-May 2023. The system at UMC was Epic (Epic Systems Corporation), queried from January 2011 to July 2023. The system queried at the VA was Computerized Patient Record System (Department of Veterans Affairs) for the period of 2017 to 2023. De-identified data collected as classified in EHRs included sex, age (in years), race and ethnicity, insurance, year of test order, test result and diagnosis code (based on International Classification of Diseases 9 or 10) associated with the lab order. The diagnostic tests used were not consistently included in EHRs, but included *T. cruzi* IgG, IgM, ELISA, and indirect immunofluorescence assays. Since the data was de-identified, linking tests at the individual patient level to differentiate initial screening and confirmatory testing was not feasible. Hospitals can use any lab but in New Orleans samples are typically sent out to ARUP, Quest, and Mayo Clinic.

Recruitment of Healthcare Professionals and Surveys
Healthcare professionals were recruited from TMC, Tulane Lakeside Hospital, UMC, Children's Hospital of New Orleans, Ochsner Medical Center, VA, East Jefferson General Hospital, DePaul Community Health Centers, Tulane School of Medicine, and Louisiana State University New Orleans School of Medicine. Department chairs were contacted about the study, and they sent an e-mail to healthcare providers, through department listservs, of the lecture and study invitation. Attendees, in person or online, received continued medical education credit from their institutions when available. Participants completed online surveys at the beginning of the training (baseline) and after it; the questions used in both surveys were the same but arranged in a different order in the post-training survey (eTable). A set of nine closed questions, previously used in US studies or developed by the Centers for Disease Control and Prevention for assessing knowledge of Chagas disease, were selected, and adapted for the study.[1-6] A set of questions were customized for each medical specialty. The survey instrument was reviewed by seven expert physicians in internal medicine and pediatrics, who also provided input in the selection of questions for each medical specialty. Additional data collected included whether prior specific education was received and demographics information. Responses were collected and stored using Qualtrics (version August 2023, Qualtrics, Provo, UT). Responses from baseline and after training were linked using a

random identification number, automatically generated for each study participant at completion of the baseline survey. To ensure data completeness, up to two reminders were sent to attendees to complete the post-assessment survey.

Educational Training

The hybrid (in person/online) training was a 30–40-minute lecture, given by two expert researchers. The training topics included epidemiology of Chagas disease, especially in the US, signs and symptoms of disease, risk factors, diagnostic testing for populations at risk and importance of early treatment with benznidazole or nifurtimox. The training was followed by a 20-minute session of question and answer. The sessions were recorded and transcribed to document questions and feedback from attendees, and then reviewed for recurring themes.

Data Analysis

Survey responses were cleaned, merged, and analyzed using R (version 4.3.1; R Core Team, 2023). Frequencies were computed to summarize demographics information of patients who had a Chagas disease test ordered and for those who screened positive. Race and ethnicity of patients screened was collected from EHR. The race classifications were: Asian, Black or African American, Hispanic or Latino, missing information, multiple, White or Caucasian, and other race; the category other race was reported in EHR but not defined. The ethnicity classifications were: asked but no answer, Hispanic or Latino, missing information, not Hispanic or Latino, and refused to report.

Frequencies were also computed to summarize physician demographics and their knowledge of Chagas disease at baseline and after training. Survey responses of *I don't know* were treated as incorrect answers. The proportion of correct responses to each question were compared from before to after training using the McNemar's chi-square test, and a significance level of 5%. A continuity correction was used for all questions except the following: presentation of chronic disease, EKG findings of cardiomyopathy, signs of infection in newborns and treatment in congenital cases. Only the 154 participants with linked baseline and post surveys were included for a more rigorous analysis, but analysis of the complete data set provided similar results at baseline and after training.

We also tested the association between prior training on Chagas disease, medical specialty, race and ethnicity of participants, experience level ($\leq$10 and >10 years of practice), patient population and knowledge. Health provider race and ethnicity was self-reported, based on classifications from the US Department of the Interior. The categories were American Indian or Alaska Native, Asian, Black or African American, Hispanic or Latino, Native Hawaiian or other Pacific Islander and White or Caucasian; the category prefer not to answer was added. The chi-square test was used to compare the global proportion of correct answers in groups stratified according to these variables. For the analysis of participant race and ethnicity, those who responded *Prefer not to answer* were excluded. For the analysis of the percentage of their patients of Hispanic/Latino origin, those who responded *I don't know* were excluded.

© 2024 Bernabé KJ et al. *JAMA Network Open*

eReferences

1. Stimpert KK, Montgomery SP. Physician awareness of Chagas disease, USA. *Emerging infectious diseases*. May 2010;16(5):871-2. doi:10.3201/eid1605.091440
2. Verani JR, Montgomery SP, Schulkin J, Anderson B, Jones JL. Survey of obstetrician-gynecologists in the United States about Chagas disease. *Am J Trop Med Hyg*. Oct 2010;83(4):891-5.
3. Edwards MS, Abanyie FA, Montgomery SP. Survey of Pediatric Infectious Diseases Society Members About Congenital Chagas Disease. *Pediatr Infect Dis J*. Jan 2018;37(1):e24-e27.
4. CDC. Chagas Disease in the U.S. What United States Health Care Providers Need To Know About Chagas Disease. Updated August 15, 2022. Accessed November 1, 2021, https://www.cdc.gov/parasites/cme/chagas/index.html
5. CDC. Chagas Disease: Optimizing Care for Pregnant Women and Children. Updated May 31, 2023. Accessed November 1, 2021, https://www.cdc.gov/parasites/cme/chagas_congenital/course.html
6. Pacheco GJ. *Chagas disease awareness amongst Texas physicians*. University of Texas School of Public Health; 2018. https://digitalcommons.library.tmc.edu/uthsph_dissertsopen/8/

© 2024 Bernabé KJ et al. *JAMA Network Open*

eTable. Survey questions

Questions	Answer choices
Have you previously heard about Chagas disease?	Yes<br>No
Where did you hear about Chagas disease?	[free text]
Have you taken a course or received a lecture specifically on Chagas disease?	Yes<br>No
What course or lecture on Chagas disease did you complete?	[free text]
Basic epidemiology	
Chagas disease is caused by a	(1) Bacterium<br>(2) Virus<br>(3) **Parasite**<br>(4) Fungus<br>(5) I don't know.
In the United States, the common transmission routes of Chagas disease are via the vector, congenital, organ/tissue transplantation, and blood transfusion.	(1) **True**<br>(2) False<br>(3) I don't know.
In what parts of the world is there transmission of the pathogen that causes Chagas disease?	(1) East Asia and parts of Africa<br>(2) Cuba, Puerto Rico, and parts of Mexico<br>(3) **Southern United States, Mexico, Central America, and South America**<br>(4) Central America, Mexico, and parts of Africa<br>(5) I don't know.
Which of the following best describes the clinical course of Chagas disease?	(1) Acute for several weeks, then immediately chronic (symptomatic)<br>(2) **Acute for several weeks, asymptomatic for years to decades then chronic (symptomatic) in a portion of infected persons**<br>(3) There are no symptoms.<br>(4) I don't know.
Patients with chronic Chagas disease may develop which of the following?	(1) Cardiac conduction abnormalities and cardiomyopathy only<br>(2) Megaesophagus and megacolon only<br>(3) **Both cardiac and gastrointestinal manifestations at the same time**<br>(4) I don't know.
Reactivation disease (recurrence of acute symptoms) is a concern for patients who:	(1) Are chronically infected and are receiving immunosuppressive treatment because of organ transplantation<br>(2) Are chronically infected and have HIV/AIDS<br>(3) Are chronically infected and acquire COVID-19<br>(4) **First and second choices only**<br>(5) All of the above<br>(6) I don't know.
**Basic epidemiology: cardiac-specific**	
Patients with chronic Chagas disease can present with which of the following?	(1) Asymptomatic<br>(2) Palpitations and shortness of breath<br>(3) Sudden death<br>(4) Prolonged constipation and abdominal pain<br>(5) **Any of the above**<br>(6)I don't know.

© 2024 Bernabé KJ et al. *JAMA Network Open*

Questions	Answer choices
**Basic epidemiology: cardiac-specific**	
Typically, the earliest manifestations of chronic Chagas cardiomyopathy include:	(1) Enlarged heart<br>(2) **Conduction system defects**<br>(3) Pericardial effusion<br>(4) I don't know.
What are EKG findings typical of Chagas cardiomyopathy?	(1) Right bundle branch block<br>(2) Left anterior fascicular block<br>(3) First degree AV block<br>(4) First and third choices only<br>(5) **All of the above**<br>(6) I don't know.
**Tools to diagnose and treat Chagas disease**	
What laboratory test should be used to diagnose Chagas disease?	(1) Blood smear<br>(2) PCR assays<br>(3) Serologic tests (antibodies to *T. cruzi* antigens)<br>(4) First and third choices only<br>(5) **All of the above**<br>(6) I don't know.
What are the important elements of the clinical evaluation of a newly diagnosed patient with chronic Chagas disease who is asymptomatic?	(1) Complete physical examination, CBC and chemistry panel (basic metabolic panel and liver function tests), radiograph of the chest<br>(2) Complete physical examination, EKG with 30-second rhythm strip, radiograph of the chest<br>(3) **Complete physical examination, CBC and chemistry panel (basic metabolic panel and liver function tests), EKG with 30-second rhythm strip, family history, detailed history of potential exposures to pathogen**<br>(4) Complete physical examination, EKG with 30-second rhythm strip, barium swallow<br>(5) I don't know.
You should request commercial laboratory diagnostic serology tests, initiate a clinical evaluation, and conduct a thorough history in a pregnant woman who/ in a patient who:	(1) Has tested positive during a blood donation, or has a sibling or mother who is Chagas positive<br>(2) Was exposed or potentially exposed to a vector in the US, Mexico, Central America or South America, or other transmission route<br>(3) Presents with onset of cardiac disease manifestations that are compatible with chronic Chagas cardiomyopathy<br>(4) **All of the above**<br>(5) I don't know.
Infected mothers should be:	(1) Treated with antiparasitics during pregnancy<br>(2) **Not treated with antiparasitics until after delivery and breastfeeding**<br>(3) Treated only if breastfeeding is planned<br>(4) Not treated, chronically infected women are not at risk for disease<br>(5) I don't know.
Should patients with chronic Chagas disease be treated with benznidazole and nifurtimox?	(1) No, there is no evidence that these drugs for Chagas disease can be effective.<br>(2) Yes, only patients younger than 5 years of age should be treated for chronic Chagas disease.

© 2024 Bernabé KJ et al. *JAMA Network Open*

Questions	Answer choices
	(3) **Treatment is always recommended for patients up to 18 years of age and generally recommended for patients aged 18 to 50**.
	(4) Only Chagas disease patients manifesting with moderate to severe cardiomyopathy.<br>(5) I don’t know.
**Congenital transmission**	
The risk of congenital transmission from a mother with chronic Chagas disease to her infant is:	(1) <1%<br>(2) **1-10%**<br>(3) 11-25%<br>(4) 26-50%<br>(5) >50%<br>(6) I don’t know.
When Chagas disease is suspected in a pregnant patient, testing should be performed on the	(1) Mother only<br>(2) Newborn only<br>(3) Mother and newborn<br>(4) **Mother, newborn, and any other child of the mother**<br>(5) I don’t know.
Clinical manifestations of congenital Chagas disease may include each of the following except:	(1) Preterm delivery<br>(2) Fetal hydrops<br>(3) Respiratory failure<br>(4) Cardiac failure<br>(5) Hepatosplenomegaly<br>(6) **Periostitis**<br>(7) Meningoencephalitis<br>(8) I don’t know.
Signs of congenital infection in newborn infants include which of the following?	(1) Prematurity or low birth weight for gestational age<br>(2) Low Apgar scores<br>(3) Hepatosplenomegaly<br>(4) Anemia<br>(5) **All of the above**<br>(6) I don’t know.
The diagnosis of congenital Chagas disease can be made by:	(1) Detection of *T. cruzi* in cord blood or peripheral blood from a newborn by the micromethod<br>(2) Detection of *T. cruzi* DNA by PCR in blood from the newborn<br>(3) Positive serologic tests for *T. cruzi* when the infant is 9-12 month of age<br>(4) **Any of the above**<br>(5) I don’t know.
When an infant is being evaluated for findings such as hepatosplenomegaly and petechiae, which diseases should be considered in the differential?	(1) Toxoplasmosis<br>(2) Syphilis<br>(3) Rubella<br>(4) Cytomegalovirus<br>(5) Herpes simplex virus<br>(6) Chagas disease<br>(7) **All of the above**<br>(8) I don’t know.
Infants with confirmed congenital Chagas disease should be treated with either nifurtimox or benznidazole	(1) **As soon as possible to improve chance of cure**<br>(2) When they are 10 years old, after the infection has become chronic<br>(3) Never, treatment is not effective for congenital Chagas disease

© 2024 Bernabé KJ et al. *JAMA Network Open*

	(4) I don't know.
**Demographics information** (select one answer)	
Type of healthcare provider	(1) Medical student<br>(2) Medical resident<br>(3) Medical fellow
**Questions**	**Answer choices**
	(4) Practicing physician<br>(5) Registered nurse<br>(6) Nurse practitioner<br>(7) Other
**Demographics information** (select one answer only)	
How many years have you been a practicing physician, registered nurse, or nurse practitioner?	(1) Less than 3 years<br>(2) 3 to 5 years<br>(3) 5 to 10 years<br>(4) More than 10 years
What is your position or role?<br>(for other healthcare professional)	[free text]
What is your primary specialization? (for medical resident, medical fellow, practicing physician, registered nurse, nurse practitioner)	[free text]
What is your secondary specialization? (for medical resident, medical fellow, practicing physician, registered nurse, nurse practitioner)	[free text]
What is your race/ethnicity?<br>(Select one or more options)	(1) White/Caucasian<br>(2) Black/African American<br>(3) American Indian or Alaska Native<br>(4) Asian<br>(5) Native Hawaiian or other Pacific Islander<br>(6) Hispanic/Latino<br>(7) Prefer not to answer
Patients of Hispanic, Latino or Spanish origin constitute what proportion of your patient population?	(1) <1%<br>(2) 1-10%<br>(3) 11-25%<br>(4) 26-50%<br>(5) >50%<br>(6) I don't know.

Correct answers to knowledge questions are shown in bold.
