# Supplementary material for: Clinician Knowledge of Chagas Disease After an Educational Intervention
Source: JAMA Netw Open. 2024 Jul 2;7(7):e2419906. doi: 10.1001/jamanetworkopen.2024.19906 (PMC11220560; doi:10.1001/jamanetworkopen.2024.19906)
Supplement: Supplement 2. — Data Sharing Statement [file jamanetwopen-e2419906-s002.pdf]

Data Sharing Statement

Bernabé. Clinician Knowledge of Chagas Disease After an Educational Intervention. *JAMA Netw Open*. Published July 02, 2024. doi:10.1001/jamanetworkopen.2024.19906

Data
Data available: Yes
Data types: Deidentified participant data
How to access data: Data can be made available upon request
When available: With publication

Supporting Documents
Document types: None

Additional Information
Who can access the data: anyone requesting the data
Types of analyses: for any purpose
Mechanisms of data availability: Data can be made available upon request
